# Persistent domestic circulation of African swine fever virus in Tanzania, 2015–2017

**DOI:** 10.1186/s12917-020-02588-w

**Published:** 2020-10-01

**Authors:** Clara M. Yona, Merijn Vanhee, Edgar Simulundu, Mariam Makange, Hans J. Nauwynck, Gerald Misinzo

**Affiliations:** 1 SACIDS Foundation for One Health, SACIDS Africa Centre of Excellence for Infectious Diseases, Sokoine University of Agriculture, Morogoro, Tanzania; 2 Department of Biosciences, Solomon Mahlangu College of Science and Education, Sokoine University of Agriculture, Morogoro, Tanzania; 3 Department of Biotechnology, VIVES University College, Roeselare, Belgium; 4 Department of Disease Control, School of Veterinary Medicine, University of Zambia, Lusaka, Zambia; 5 Department of Veterinary Microbiology, Parasitology and Biotechnology, College of Veterinary Medicine and Biomedical Sciences, Sokoine University of Agriculture, Morogoro, Tanzania; 6 Laboratory of Virology, Faculty of Veterinary Medicine, University of Gent, Merelbeke, Belgium

**Keywords:** African swine fever, African swine fever virus, *Asfarviridae*, genotype, *Sus scrofa*, Tanzania

## Abstract

**Background:**

African swine fever (ASF) is a highly fatal viral hemorrhagic disease of domestic pigs that threatens livelihoods and food security. In Africa, ASF virus (ASFV) circulates in sylvatic (transmission between warthogs and soft argasid ticks) and domestic (transmission between domestic pigs) cycles, with outbreaks resulting from ASFV spill-over from sylvatic cycle. A number of outbreaks were reported in different parts of Tanzania between 2015 and 2017. The present study investigated ASFV transmission patterns through viral DNA sequencing and phylogenetic analysis. A total of 3120 tissue samples were collected from 2396 domestic pigs during outbreaks at different locations in Tanzania between 2015 and 2017. Partial sequencing of the *B646L* (p72) gene was conducted for diagnostic confirmation and molecular characterization of ASFV. Phylogenetic analysis to study the relatedness of current ASFV with those that caused previous outbreaks in Tanzania and representatives of all known 24 ASFV was performed using the Maximum Composite Likelihood model with 1000 bootstrap replications in MEGA 6.0.

**Results:**

ASFV was confirmed to cause disease in sampled domestic pigs. ASFV genotypes II, IX, and X were detected from reported outbreaks in 2015–2017. The current ASFV isolates were similar to those recently documented in the previous studies in Tanzania. The similarities of these isolates suggests for continuous circulation of ASFV with virus maintenance within the domestic pigs.

**Conclusions:**

Genetic analysis confirmed the circulation of ASFV genotypes II, IX, and X by partial *B646L* (p72) gene sequencing. The similarities of current isolates to previously isolated Tanzanian isolates and pattern of disease spread suggest for continuous circulation of ASF with virus’ maintenance in the domestic pigs. Although certain viral genotypes seem to be geographically restricted into certain zones within Tanzania, genotype II seems to expand its geographical range northwards with the likelihood of spreading to other states of the East African Community. The spread of ASFV is due to breach of quarantine and transportation of infected pigs via major highways. Appropriate control measures including zoosanitary measures and quarantine enforcement are recommended to prevent ASF domestic circulation in Tanzania.

## Background

African swine fever (ASF) is a contagious viral hemorrhagic disease of pigs affecting domestic pigs and wild pigs [1]. African swine fever is endemic in sub-Saharan countries and the mortality rates can reach up to 100% [2]. African swine fever is caused by ASF virus (ASFV), a DNA arbovirus belonging to the *Asfivirus* genus and a sole member of the *Asfarviridae* family [3]. The ASFV virion is enveloped, has an icosahedral morphology and contains a double-stranded DNA genome whose size ranges between 170 and 193 kilo base pairs depending on the isolate [4]. Warthogs are reservoir hosts that are persistently infected with no obvious clinical disease, and soft ticks of the genus *Ornithodoros* act as vectors of ASFV and contribute to viral maintenance within the sylvatic cycle as well as in transmitting the virus to domestic pigs [5]. Transmission of ASFV from the sylvatic cycle to domestic pigs occurs through a tick bite, feeding contaminated warthog carcasses to domestic pigs and/or contact with warthog faeces [6]. Once ASFV is transmitted to domestic pigs, the virus spreads between domestic pigs through contact between infected and susceptible pigs, feeding pigs with meat or via fomites such as contaminated clothing, shoes, equipment and vehicles [7].

The existence of the sylvatic cycle contributes to a rich genetic diversity of ASFV. Based on partial amplification and sequence analysis of the p72 (*B646L*) gene, 24 genotypes of ASFV have been identified [8–10]. All of the 24 ASFV genotypes have been described in African countries, South of the Sahara, 23 of which are currently restricted to Eastern and Southern Africa [2, 9]. Genotypes I, II and IX of ASFV have been reported to spread beyond their traditional geographical range. For instance, genotype I spread from West Africa to Europe, South America and the Caribbean [11]. On the other hand, genotype II, which was known to circulate in Zambia, Malawi, and Mozambique, spread to the Caucasus and afterward to the European Union, Russia and China [12–16]. Furthermore, genotype II ASFV has been introduced to Tanzania and Zimbabwe, where it was never known to circulate [17, 18]. Similarly, genotype IX which is restricted to Eastern Africa has been reported to spread to Western Africa [19].

The spread of ASFV beyond African countries south of the Sahara and its traditional geographical boundaries poses a threat to the global pig industry, international trade market and food security. In 2018, the ASFV spread to China, a major pork producer, and afterward, the virus has spread to Asian countries including Mongolia, Vietnam, Indonesia, Democratic People’s Republic of Korea, Lao People’s Democratic Republic, Myanmar, The Philippines, Republic of Korea, Timor-Leste and Cambodia [14–16].

A number of sporadic ASF outbreaks have been reported since 2000 in different parts of Tanzania, associated with ASFV genotypes II, IX, X, XV and XVI [17, 20–23]. There appears to be a geographical restriction of the ASFV genotypes in Tanzania with genotype II being restricted to Southwestern Tanzania, genotype IX to Northwestern Tanzania, genotypes X and XVI to Northeastern Tanzania and genotype XV to Eastern Tanzania [17, 20–22, 24]. These outbreaks in other parts of Tanzania end up in Dar es Salaam due to the transportation of infected pigs for sale and slaughter from other parts of the country to this main commercial capital [20, 21]. Many outbreaks have been reported in different parts of Tanzania between 2010 and 2017. The aim of this study was to investigate the ASFV transmission patterns through virus genotyping in order to understand the relationship between ASF outbreaks.

## Results

### Clinical signs and postmortem findings

**Fig. 1:**
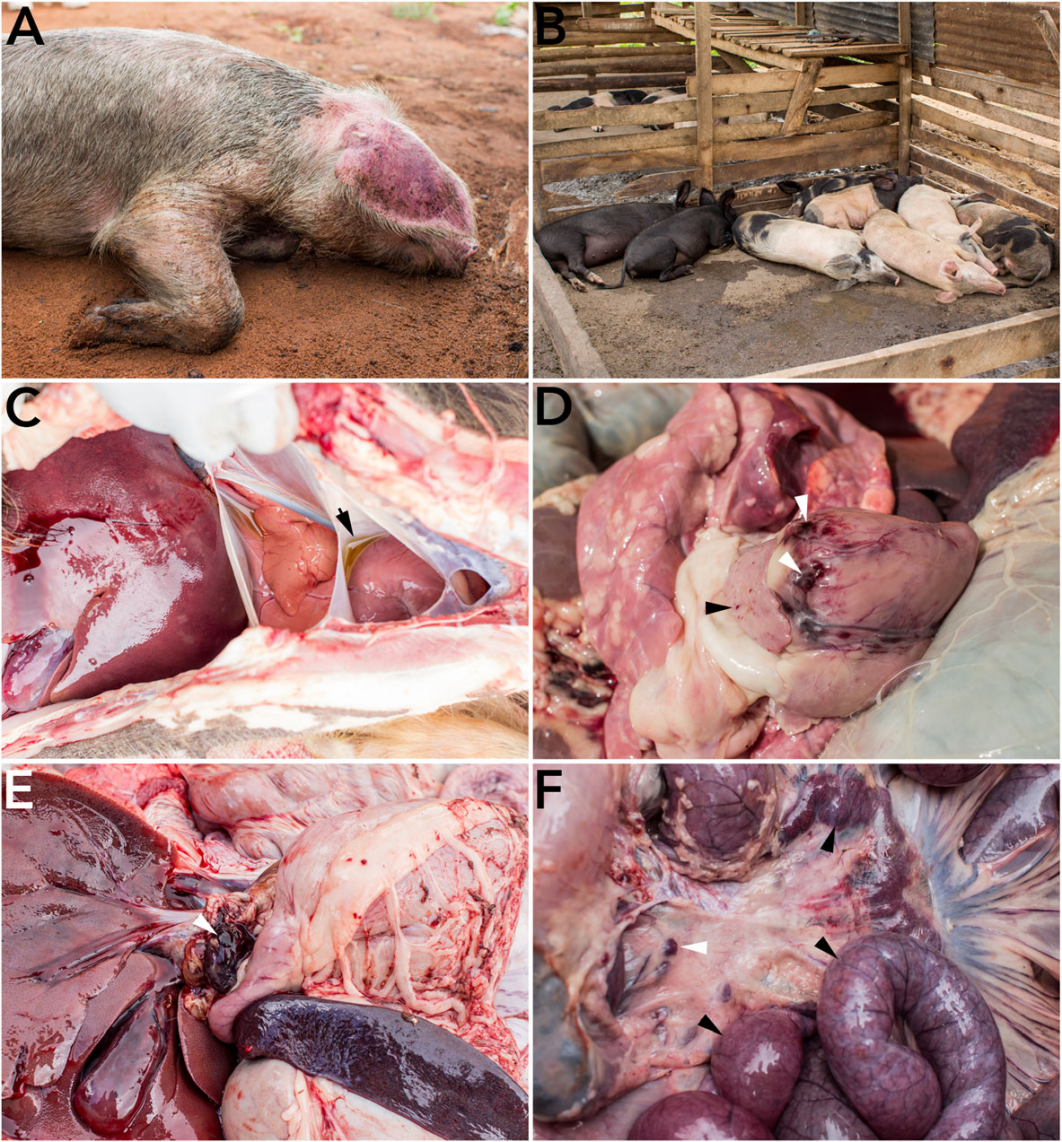
Clinical signs and postmortem findings observed in domestic pigs with African swine fever. **a** Ventral recumbence and cutaneous congestion especially on the outer side of pinna and **b** loss of appetite, lateral recumbence and a tendency to stay together at one side of the pen were observed in pigs with African swine fever. At postmortem, **c** the pericardial and thoracic cavities were filled with straw tinged fluid (indicated by an arrow), **d** hemorrhages of the heart (indicated by an arrow) especially at the atrioventricular junctions, **e** hemorrhages of the gastrohepatic lymph node (indicated by an arrow) and **f** enteritis and hemorrhages of the mesenteric lymph nodes (indicated by an arrow head)

Clinical signs observed in sick pigs included a high fever (> 40 °C), anorexia, staggering gait, shivering and cutaneous congestion particularly on the outer side of the pinna, belly, limbs and genitalia (Fig. 1a). Pigs were dull and stayed together at one side of their pens (Fig. 1b). Abortion was observed in pregnant sows. At postmortem, the pericardial and thoracic cavities were filled with straw tinged fluid (Fig. 1c). In addition, postmortem findings included hemorrhages in the spleen, heart, kidneys and lymph nodes especially the gastrohepatic, thoracic, mesenteric and renal lymph nodes (Fig. 1d and f). Splenomegaly (enlargement of the spleen) and enteritis were also observed (Fig. 1e).

### Confirmatory diagnosis of ASFV

In total, 3120 tissues samples collected from different parts of Tanzania were screened using ASF diagnostic PCR. Analysis of collected tissue samples confirmed the presence of ASFV in 2170 tissue samples (Table 1). The PCR products of ASFV nucleic acid with a band size of 257 base pairs using primers PPA1 and PPA2 were obtained.

**Table 1:** Epidemiological information from farms that were affected with ASFV outbreaks between 2015 and 2017 in Tanzania

Location	Distance (km)^a^	Region	Outbreak month	Morbidity	Mortality	Herd size	Apparent case fatality (%) rate	No. of pigs sampled	No. of positive pigs	Type of operation	Breed	Age affected	Management system
Mwanza	5	Mwanza	Dec-15	480	480	700	100	200	200	Commercial farm	Cross	All age groups	Intensive system
Manyoni	4	Singida	May-15	80	68	100	85	65	55	Backyard farm	Cross	All age groups	Intensive system
Kigoma	3	Kigoma	May-15	58	40	78	69	40	29	Backyard farm	Cross	All age groups	Intensive system
Bukoba	12	Kagera	Dec-15	90	76	136	84	54	51	Backyard farm	Exotic	All age groups	Semi-intensive system
Magu	3	Mwanza	Jul-16	100	94	130	94	50	50	Backyard farm	Cross	All age groups	Intensive system
Ngara	4	Kagera	Jul-16	50	39	98	78	30	30	Backyard farm	Cross	Adults	Intensive system
Babati	3	Manyara	Aug-16	57	42	87	74	38	38	Backyard farm	Local	All age groups	Intensive system
Mbarali	5	Mbeya	Mar-16	123	118	150	96	112	110	Backyard farm	Cross	All age groups	Intensive system
Tukuyu	2	Mbeya	Feb-16	150	135	160	90	120	70	Commercial farm	Cross, Exotic	All age groups	Intensive system
Uyole	2	Mbeya	Feb-16	65	60	87	92	30	30	Backyard farm	Cross	All age groups	Intensive system
Kalambo	46.7	Rukwa	Mar-17	163	140	195	86	120	110	Backyard farm	Local	All age groups	Free ranging system
Ileje	74.3	Mbeya	Mar-17	35	33	58	94	33	33	Backyard farm	Cross	Adults	Intensive system
Mbozi	8	Mbeya	Mar-17	350	348	370	99	100	87	Commercial farm	Cross	All age groups	Intensive system
Kongwa	16	Dodoma	May-17	167	167	180	100	160	100	Commercial farm	Cross	All age groups	Intensive system
Dodoma	14	Dodoma	May-17	120	120	130	100	110	80	Backyard farm	Cross	All age groups	Intensive system
Mpwapwa	58	Dodoma	May-17	340	320	500	94	200	194	Backyard farm	Cross	All age groups	Intensive system
Gairo	1	Dodoma	Apr-17	140	127	165	91	98	95	Backyard farm	Cross	All age groups	Intensive system
Mbagala	15	Dar es Salaam	Feb-17	230	220	240	96	200	200	Backyard farm	Cross	All age groups	Intensive system
Mazimbu	5	Morogoro	May-17	1210	1210	1220	100	360	360	Commercial farm	Cross	All age groups	Intensive system
Mzumbe	7	Morogoro	May-17	300	290	340	97	100	100	Commercial farm	Cross	All age groups	Intensive system
Morogoro	6	Morogoro	May-17	69	64	76	93	56	38	Backyard farm	Exotic	All age groups	Intensive system
Kibaha	2	Pwani	May-17	234	234	400	100	120	110	Commercial farm	Cross	All age groups	Intensive system

^a^Distance of the farm where ASF outbreak occurred from the major highway

### Molecular characterization of ASFV

**Fig. 2:**
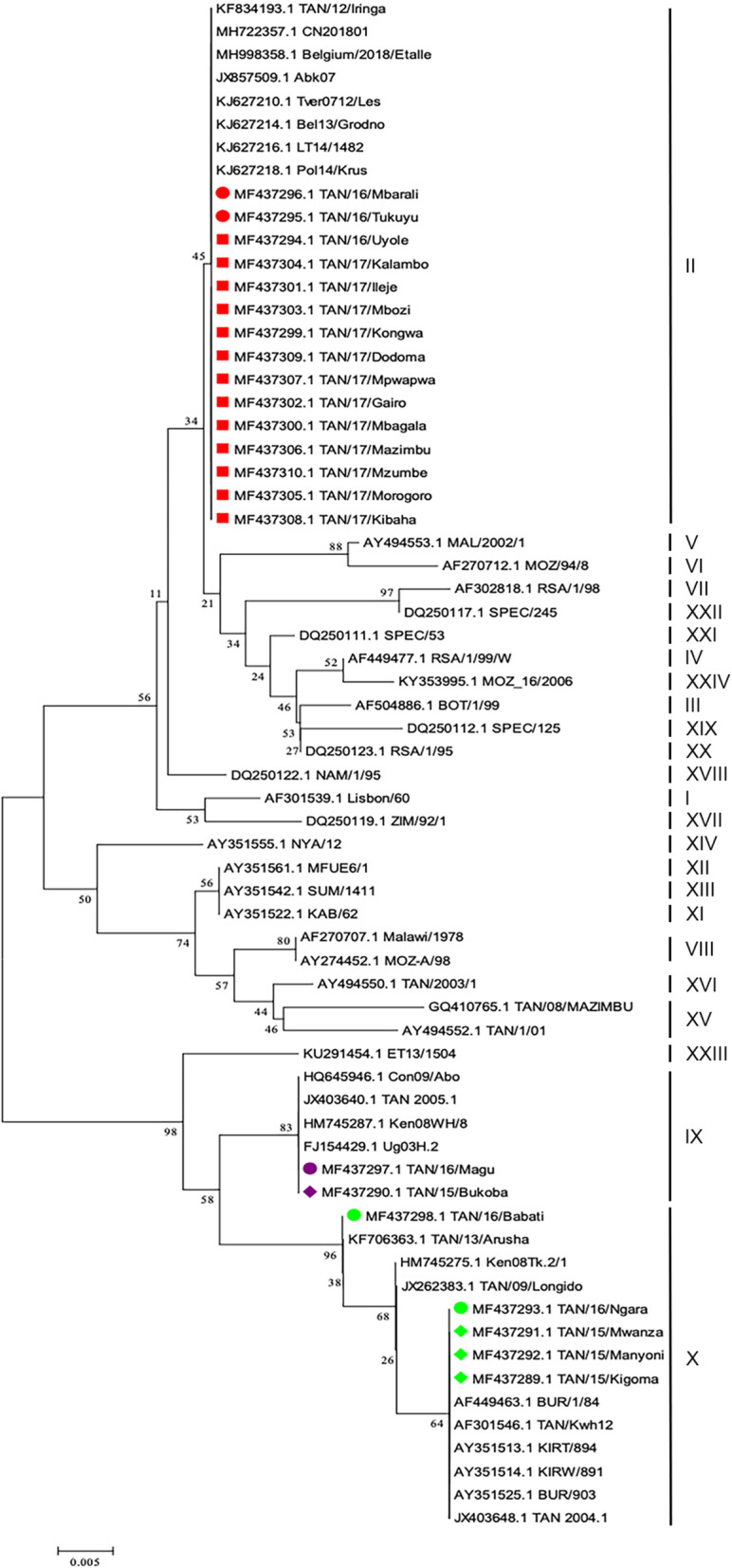
Phylogenetic relationship of African swine fever viruses (ASFV). The ASFV which were collected in the present study and from 2015 to 2017 are indicated by square, circle and diamond, respectively. Genotype II, IX, X, XV and XVI are labeled in red, purple, green, blue and pink respectively. Phylogeny was inferred following 1,000 bootstrap replications and node values show percentage bootstrap support. Scale bar indicates nucleotide substitution per site. The GenBank accession numbers for the different *B646L* (p72) gene are indicated in parenthesis

**Fig. 3:**
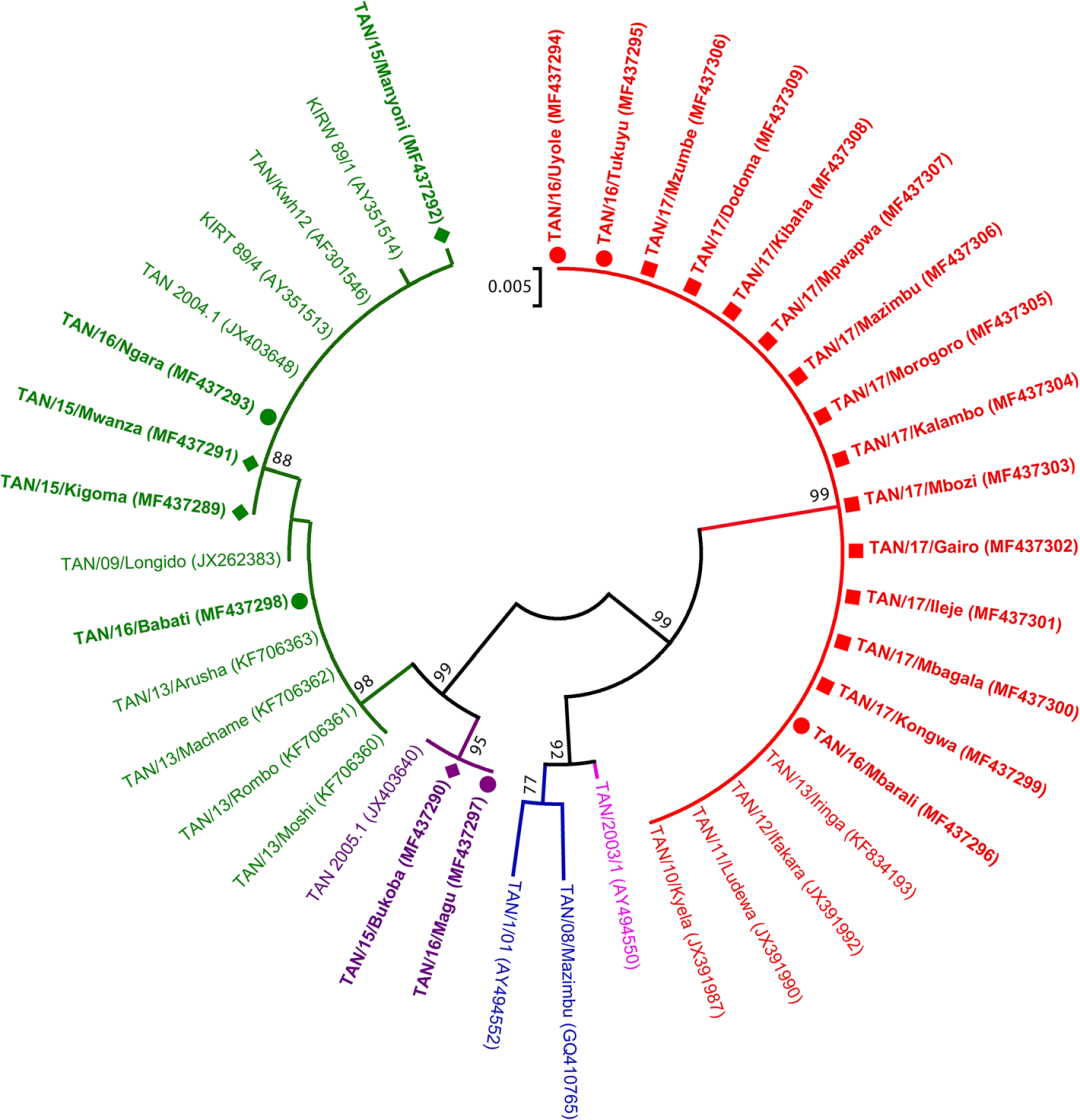
Neigbour joining phylogenetic tree of the partial *B646L* (p72) gene of Tanzanian ASFV isolates of 2015–2017 relative to representatives of all known 24 p72 genotypes indicated as I – XXIV. The evolutionary history was inferred by the Maximum Composite Likelihood model with 1000 replicates boostrap analysis. ASFV Tanzania isolates characterized in this study are marked in red, purple and green for genotype II, IX and X respectively with the square, circle and diamond signs for 2015, 2016 and 2017

**Fig. 4:**
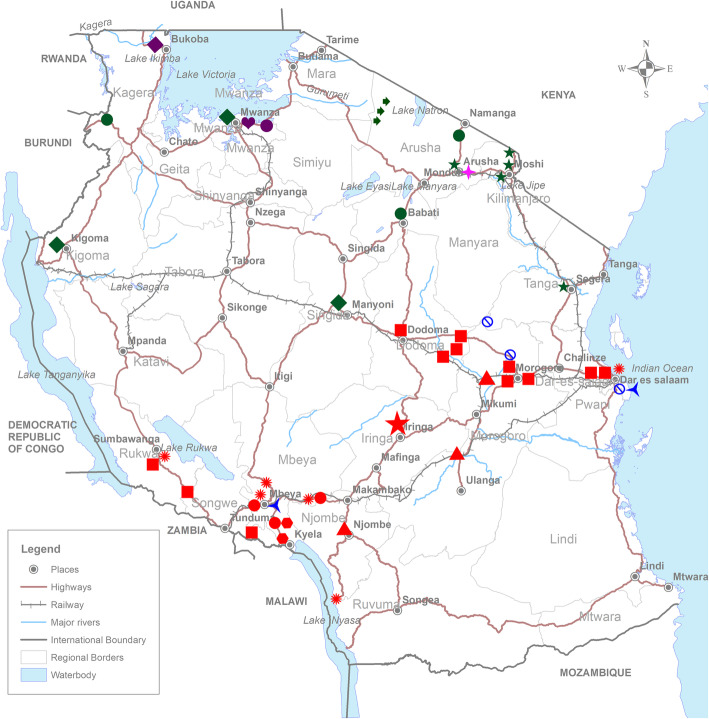
Map of Tanzania showing reported African swine fever outbreaks and ASF virus (ASFV) genotypes between 2010 and 2017. Africa swine fever outbreaks were reported in Southwestern, Eastern, Central, Northeastern and Northwestern Tanzania caused by ASFV genotypes II (red), IX (purple), X (green), XV (blue) and XVI (pink). The ASFV strains collected in Tanzania between 1968 and 2017 are indicated using different symbols; 1968 (➨), 1989 (➨), 2001 ( ), 2003 (✖), 2005 (♥), 2008 (🛇), 2010 ( ), 2011 (✺), 2012 (▲), 2013 (★), 2015 (♦), 2016 (●) and 2017 (■). The map was developed using QGIS version 3.4.4 (https://www.qgis.org/en/site/about/index.html). Afterwards, the symbols indicating ASF outbreak locations were added to the map using Adobe Photoshop CC 2017.0.0 Release, Adobe Systems Incorporated

A phylogenetic tree was constructed by the Neighbor-Joining method in order to determine the genetic relationship between the ASFV strains collected during 2015 and 2017 outbreaks, and previously sequenced Tanzanian ASFV strains available in GenBank (Fig. 2). The ASFV strains collected during this study (accession numbers MF437289 - MF437310) clustered with p72 genotypes II, IX and X (Fig. 2). Genotype II ASFV strains were characterized from Southwestern, Central and Eastern Tanzania, genotype IX from Northwestern parts of Tanzania around Lake Victoria and genotype X from Northwestern, Northeastern and Central parts of Tanzania (Fig. 4). The ASFV collected from outbreaks between 2015 and 2017 clustered into genotype II, IX and X along with ASFV that were responsible for previous outbreaks in Africa, Europe and Asia (Fig. 3).

## Discussion

Several outbreaks of a highly fatal hemorrhagic disease affecting domestic pigs, suspected to be ASF based on clinical signs and postmortem findings, were reported in different parts of Tanzania between 2015 and 2017. ASF remains a major constraints to the pig industry in Tanzania with reported outbreaks throughout the year. There is neither a cure nor vaccine to prevent ASF infection. The control and eradication measures of ASF are based on surveillance, epidemiological investigation, animal movement control, quarantine and zoosanitary measures. Early detection of the disease and its spread is important for a successful surveillance and accurate diagnostic procedures are important for effective quarantine and control measures [25]. In this study, molecular methods were used to identify and characterize ASFV from domestic pigs that died of hemorrhagic disease outbreaks in Tanzania.

The results obtained in the 2015–2017 outbreaks confirm ASF outbreaks in domestic pigs in the studied areas. In the present study, these ASF outbreaks were confirmed to be caused by ASFV belonging to genotypes II, IX and X (Figs. 2 and 3). Prior to 2015, the ASFV that caused ASF outbreaks in Tanzania clustered into genotypes II, IX, X, XV and XVI (Table 2) [20, 22–24]. The recent ASF outbreaks were caused by ASFV that were 100% genetically identical to previously reported viruses, for each of the genotype II, IX and X (Fig. 2). The identity of ASFV between previous and recent outbreaks and the pattern of disease spread strongly indicate domestic pig-to-pig transmission.

**Table 2:** List of the representative of all 24 known ASFV isolates from different locations and isolates characterized from this study used and ASFV genetically characterized in the present study based on partial *B646L* (p72) genotypes for evolutionary analysis

Host species	Isolate	Year of Isolation	Town/District	Country	GenBank Accession number	p72 genotype	Reference
Pig	TAN/10/Kyela	2010	Kyela	Tanzania	JX391987	II	[17]
Pig	TAN/11/Ludewa	2011	Ludewa	Tanzania	JX391990	II	[17]
Pig	TAN/12/Ifakara	2012	Ifakara	Tanzania	JX391992	II	[17]
Pig	TAN/13/Iringa	2013	Iringa	Tanzania	KF834193	II	Unpublished
DP	CN201801	2018	Shenbei	China	MH722357	II	[15]
WB	Belgium/2018/Etalle	2018	Etalle	Belgium	MH998358	II	[26]
DP	Abk07	2007	Gulripish	Georgia	JX857509	II	[13]
DP	Tver0712/Les	2012	Lesnoi	Russia	KJ627210	II	[13]
DP	Bel13/Grodno	2013	Grodno	Belarus	KJ627214	II	[13]
EWB	LT14/1482	2014	Alytus County	Lithuania	KJ627216	II	[13]
EWB	Pol14/Krus	2014	Kruszyniany	Poland	KJ627218	II	[13]
DP	TAN/16/Mbarali	2016	Mbarali	Tanzania	MF437296	II	This study
DP	TAN/16/Tukuyu	2016	Tukuyu	Tanzania	MF437295	II	This study
DP	TAN/16/Uyole	2016	Uyole	Tanzania	MF437294	II	This study
DP	TAN/17/Kalambo	2017	Kalambo	Tanzania	MF437304	II	This study
DP	TAN/17/Ileje	2017	Ileje	Tanzania	MF437301	II	This study
DP	TAN/17/Mbozi	2017	Mbozi	Tanzania	MF437303	II	This study
DP	TAN/17/Kongwa	2017	Kongwa	Tanzania	MF437299	II	This study
DP	TAN/17/Dodoma	2017	Dodoma	Tanzania	MF437309	II	This study
DP	TAN/17/Mpwapwa	2017	Mpwapwa	Tanzania	MF437307	II	This study
DP	TAN/17/Gairo	2017	Gairo	Tanzania	MF437302	II	This study
DP	TAN/17/Mbagala	2017	Mbagala	Tanzania	MF437300	II	This study
DP	TAN/17/Mazimbu	2017	Mazimbu	Tanzania	MF437306	II	This study
DP	TAN/17/Mzumbe	2017	Mzumbe	Tanzania	MF437310	II	This study
DP	TAN/17/Morogoro	2017	Morogoro	Tanzania	MF437305	II	This study
DP	TAN/17/Kibaha	2017	Kibaha	Tanzania	MF437308	II	This study
DP	TAN/15/Bukoba	2015	Bukoba	Tanzania	MF437290	IX	This study
DP	TAN/16/Magu	2016	Magu	Tanzania	MF437297	IX	This study
DP	Ug03H.2	2003	Hoima	Uganda	FJ154429	IX	[27]
WH	Ken08WH/8	2008	Machakos	Kenya	HM745287	IX	[19]
DP	CON09/Abo	2009	Abo, Cuvette	Rep. Congo	HQ645946	IX	[19]
DP	TAN 2005.1	2005	Mwanza	Tanzania	JX403640	IX	Unpublished
DP	TAN/16/Ngara	2016	Ngara	Tanzania	MF437293	X	This study
DP	TAN/16/Babati	2016	Babati	Tanzania	MF437298	X	This study
DP	TAN/15/Mwanza	2015	Mwanza	Tanzania	MF437291	X	This study
DP	TAN/15/Manyoni	2015	Manyoni	Tanzania	MF437292	X	This study
DP	TAN/15/Kigoma	2015	Kigoma	Tanzania	MF437289	X	This study
DP	BUR/1/84	1984	NK	Burundi	AF449463	X	[11]
WH	TAN/Kwh12	1968	Serengeti National Park	Tanzania	AF301546	X	[24]
Tk	KIRT/894	1989	Serengeti National Park	Tanzania	AY351513	X	[24]
WH	KIRW/891	1989	Serengeti National Park	Tanzania	AY351514	X	[24]
DP	BUR/903	1990	Muyinga	Burundi	AY351525	X	[24]
Tk	Ken08Tk.2/1	2008	Machakos	Kenya	HM745275	X	[19]
Tk	TAN 2004.1	2004	Kigoma	Tanzania	JX403648	X	Unpublished
WH	TAN/09/Longido	2009	Longido	Tanzania	JX262383	X	[21]
WH	TAN/13/Moshi	2013	Moshi	Tanzania	KF706360	X	[22]
DP	TAN/13/Rombo	2013	Rombo	Tanzania	KF706361	X	[22]
DP	TAN/13/Machame	2013	Machame	Tanzania	KF706362	X	[22]
DP	TAN/13/Arusha	2013	Arusha	Tanzania	KF706363	X	[22]
DP	TAN/08/Mazimbu	2008	Mazimbu	Tanzania	GQ410765	XV	[17]
DP	Tan/1/01	2001	Dar es Salaam	Tanzania	AY494552	XV	[24]
DP	Tan/2003/01	2003	Arusha	Tanzania	AY494550	XVI	[24]
DP	Lisbon/60	1960	Lisbon	Portugal	AF301539	I	[11]
WH	BOT/1/99	1999	Sherwood	Botswana	AF504886	III	[11]
WH	RSA/1/99/W	1999	Thabazimbi	South Africa	AF449477	IV	[11]
DP	MAL/2002/1	2002	Mpemba	Malawi	AY494553	V	[24]
DP	MOZ/94/8	1994	Manica	Mozambique	AF270712	VI	[28]
DP	RSA/1/98	1998	Potgietersrus	South Africa	AF302818	VII	[28]
DP	Malawi/1978	1978	NK	Malawi	AF270707	VIII	[28]
DP	MOZ-A/98	1998	Tete	Mozambique	AY274452	VIII	[28]
Tk	KAB/62	1983	Livingstone	Zambia	AY351522	XI	[24]
Tk	MFUE6/1	1982	Mfue	Zambia	AY351561	XII	[24]
Tk	SUM/1411	1983	Sumbu Park	Zambia	AY351542	XIII	[24]
Tk	NYA/12	1986	Kalumo	Zambia	AY351555	XIV	[24]
DP	TAN/1/01	2001	Dar es Salaam	Tanzania	AY494552	XV	[24]
DP	TAN/2003/1	2003	Arusha	Tanzania	AY494550	XVI	[24]
DP	ZIM/92/1a	1992	Gweru Midlands	Zimbabwe	DQ250119	XVII	[8]
DP	NAM/1/95	1995	Windhoek	Namibia	DQ250122	XVIII	[8]
DP	SPEC/125	1987	Ellisras	South Africa	DQ250112	XIX	[8]
DP	RSA/1/95	1995	Hoiedspruit	South Africa	DQ250123	XX	[8]
DP	SPEC/53	1985	Letaba	South Africa	DQ250111	XXI	[8]
DP	SPEC/245	1992	Louis Trichardt	South Africa	DQ250117	XXII	[8]
DP	ET13/1504	2013	Debre Zeit	Ethiopia	KU291454	XXIII	[9]
SF	MOZ_16/2006	2006	Gorongosa National Park	Mozambique	KY353995	XXIV	[10]

*DP* Domestic pigs, *WB* Wild boars, *EWB* European wild boars, *WH* Warthogs, *SF* Soft ticks, *NK* Not known

Prior to 2015, genotype II ASFV were reported during outbreaks in Southwestern and Eastern parts of Tanzania (Figs. 2 and 4) [17]. Genotype II ASFV is thought to have been introduced into Tanzania in Kyela, a town in Southwestern Tanzania at the Tanzania - Malawi border following an outbreak in Karonga in 2010 [17]. Since the introduction of genotype II ASFV, the virus spread within Southwestern parts of the country with occasional incursion into Eastern Tanzania [17]. In the present study, we found that genotype II ASFV continued to circulate in previously reported areas and it spread into new areas of Central Tanzania (Fig. 4). Previously, ASF outbreaks in Eastern Tanzania were linked to outbreaks in Southwestern Tanzania due to transportation of live pigs for sale in the main commercial city of Dar es Salaam [17]. The Southwestern part of the country is linked to Dar es Salaam by a major highway from Sumbawanga via Tunduma, Mbeya, Iringa and Morogoro (Fig. 4). Furthermore, the different ASF outbreaks between 2015 and 2017 due to genotype II ASFV occurred in various locations along and in the vicinity of the Morogoro - Dodoma highway, which branches off in Morogoro from the Tunduma - Dar es Salaam highway. The outbreaks involving genotype II ASFV seem to have originated from Southwestern Tanzania (Mbeya and Rukwa regions) before spreading to Dar es Salaam, Morogoro, Dodoma and Pwani regions in the vicinity of major highways. The spread of the virus along these highways could be due to illegal transportation of infected domestic pigs from areas under quarantine, as described in previous reports [17, 20].

Genotype II ASFV is highly virulent and has been reported to spread beyond its traditional geographical boundaries of Malawi, Mozambique and Zambia into Madagascar, Mauritius, Zimbabwe, Tanzania, the Caucuses region, Russia, Europe and Asia [12, 14–16, 27, 29]. The 2015–2017 Tanzanian ASFV p72 genotype II isolates clustered with ASFV p72 genotype II isolates that have been reported to cause outbreaks in Belgium (2018), Georgia (2007), Russia (2012), Belarus (2013), Lithuania (2014), Poland (2014) and China (2018) [13, 15, 26] (Fig. 3). If appropriate control measures of these genotype II viruses are not strictly enforced, we predict that this virus could possibly spread northwards and ultimately into bordering countries of Rwanda and Uganda, as these two countries are connected with Tanzania by major highways (Fig. 4). We recommend that stakeholders involved with ASF control be vigilant in order to prevent further spread of genotype II ASFV beyond Dodoma city, where it has reached.

In the present study, we found that ASFV genotype X circulated in Northeastern Tanzania, similar to other ASFV genotypes that have been previously described in the area [17, 22]. In addition, we found that genotype X ASFV has spread into new areas within Central and Western Tanzania (Fig. 4). The similarity of current ASFV to previously documented ASFV in Northeastern Tanzania indicates the continuous circulation of the virus with its maintainance in the domestic cycle. Additionally, phylogenetic analysis clustered current ASFV genotype X isolates with previously characterized ASFV isolates from Burundi (1999) and Kenya (2008) [11, 24]. The alignment of 404 nucleotide long sequence of the variable 3′-end of the *B646L* (p72) gene of ASFV Northeastern Tanzanian with 2008 ASFV outbreak in Kenya show only three nucleotide substitution (A→T, C→T, A→G) [19]. The Northern Tanzania is characterized with presence of wildlife protected areas. In East and Southern Africa, the ancient sylvatic cycle have been reported to play part in the epidemiology of the disease [5, 19, 24].

The ASFV genotype IX was confirmed to cause ASF outbreaks in Northwestern Tanzania. It was observed that ASFV genotype IX is restricted to Northwestern Tanzania, as it was 100% similar to ASFV isolates that caused ASF outbreaks in 2005 in Mwanza, Tanzania. Additionally, phylogenetic analysis revealed that the ASFV IX isolates were closely related to ASFV characterized in Uganda (2003), Kenya (2008) and Democratic Republic of Congo (2009) [19, 27]. ASFV sporadic outbreaks in Northwestern Tanzania is highly likely due to uncontrolled movement of pigs and pig products from affected areas to unaffected areas. However, studies that focus on ASF outbreaks investigation between neighboring countries should be encouraged for understanding the potential source of such viruses, variation and extent.

The isolation of ASFV from domestic pigs reports the circulation of these viral genotypes in the domestic pig population in Tanzania. However, this study points up for further isolation and epidemiological investigation in order to fully understand the variations, extent and potential sources of current ASF outbreaks in the region. The occurrence and spread of ASF between different parts of Tanzania is likely due to breach of quarantine imposed in areas affected with ASF. It is mostly likely that pig traders smuggle and transport pigs or pig meat from areas affected with ASF, where the prices are lower, into unaffected areas. Poor biosecurity measures in affected farms and slaughter slabs and swill feeding increase the likelihood of ASFV spread at a given locality, as has been previously described [17, 22]. Transportation of pig and pig products for regional market should be controlled to prevent ASFV spreading to other states of the East African Community, as ASFV genotype II has previously known to spread beyond its geographical range.

## Conclusions

This study confirmed that ASFV genotype II, IX, and X were responsible for the reported outbreaks between 2015 and 2017. The similarities of the current Tanzanian ASFV isolates with those recently documented in the previous studies and pattern of spread in adjacent location during outbreaks suggest the continuous circulation of ASF with the virus’s maintenance within the domestic cycle. Although certain viral genotypes seem to be geographically restricted into certain zones within Tanzania, genotype II seems to expand its geographical range northwards with the likelihood of spreading to other states of the East African Community. The spread of ASFV was mapped along major highways in Tanzania; this is likely due to the uncontrolled movement of pigs from affected to unaffected areas, breach of quarantine and poor zoosanitary measures. This study recommends continuous virus isolation and investigation to understand the epidemiology of ASFV in Tanzania and neighboring countries for local and inter-regional effective control and prevention interventions.

## Methods

### Study area

Samples were collected from domestic pigs following reports of suspected ASF outbreaks in different locations within Tanzania between 2015 and 2017. Samples were collected from Mwanza, Manyoni, Kigoma, and Bukoba districts in the year 2015, Babati, Ngara, Magu, Mbeya Municipality, Rungwe and Mbarali districts in the year 2016 and Kalambo, Ileje, Mbozi, Kongwa, Dodoma, Mpwapwa, Gairo, Mbagala, Mvomero, Morogoro Municipality and Kibaha districts in the year 2017 as indicated in Table 1.

### Sample collection and processing

A total of 3120 tissue samples were collected from 2396 domestic pigs that died from a hemorrhagic disease typical of ASF. Epidemiological information from these farms with outbreaks were collected. Clinical observation of pigs was performed prior to sampling. Tissue samples including spleen, mesenteric lymph nodes, lungs and kidney were collected from dead domestic pigs from suspected ASF. Tissues were temporarily stored at -20 °C before they were transported in ice cool boxes to the laboratory. Approximately, 1 g of each tissue sample was homogenized in 3 mL of sterile phosphate-buffered saline (PBS), followed by centrifugation of the homogenate at 6000 *g* for five minutes at 4 °C. The tissue supernatant was transferred into a cryovial and stored at -80 °C until DNA extraction.

### Detection of ASF in pig samples

Aliquots (100 µL) of each of the homogenized tissue samples from the same pig were pooled before conducting DNA extraction. DNA was extracted from the supernatant of pooled homogenized tissues using a QIAamp nucleic acid extraction kit (Qiagen, Hilden, Germany), following the manufacturer’s instructions. The presence of ASFV DNA was detected by polymerase chain reaction (PCR) using the ASF diagnostic primer set PPA1 (5′-AGT TAT GGG AAA CCC GAC CC-3′) and PPA2 (5′-CCC TGA ATC GGA GCA TCC T-3′) that partially amplify the *B646L* (p72) gene as previously described by Aguero et al. [30].

### Genetic characterization of ASFV

Genetic characterization of ASFV was conducted in samples confirmed with ASFV by partial nucleotide amplification of the *B646L* (p72) gene using primers p72U (5′-GGC ACA AGT TCG GAC ATG T-3′) and p72D (5′-GTA CTG TAA CGC AGC ACA G-3′) as previously described by Bastos et al. [11]. Afterwards, the PCR products were subjected to automated dideoxynucleotide cycle sequencing using BigDye Terminator Cycle sequencing kit version 3.1 (Applied Biosystems, Foster City, CA) and generated chromatograms were read by Sequence Scanner version 1.0 software (Applied Biosystems, Foster City, CA). The obtained nucleotide sequences were submitted to GenBank and were afterwards assigned with GenBank accession numbers (Table 2). The similarity search of the obtained nucleotide sequences against other ASFV sequences at GenBank database was performed using BLASTN version 2.6.0. The ASFV nucleotide sequences were aligned with previously characterized Tanzanian and global known 24 ASFV genotypes’ nucleotide sequences available at GenBank using ClustalW. Phylogenetic analysis was performed using the Neighour-Joining method with 1000 bootstrap replications. The evolutionary history was inferred by the Maximum Composite Likelihood model using MEGA 6.0 [31].

## Data Availability

The datasets generated and/or analysed during the current study are available at GenBank https://www.ncbi.nlm.nih.gov/popset/1463932638. The accession numbers are presented in Table 2.
